# Multi‐institutional validation of a novel textural analysis tool for preoperative stratification of suspected thyroid tumors on diffusion‐weighted MRI

**DOI:** 10.1002/mrm.25743

**Published:** 2015-05-20

**Authors:** Anna M. Brown, Sidhartha Nagala, Mary A. McLean, Yonggang Lu, Daniel Scoffings, Aditya Apte, Mithat Gonen, Hilda E. Stambuk, Ashok R. Shaha, R. Michael Tuttle, Joseph O. Deasy, Andrew N. Priest, Piyush Jani, Amita Shukla‐Dave, John Griffiths

**Affiliations:** ^1^ Cancer Research UK Cambridge Institute, University of CambridgeLi Ka Shing CentreRobinson WayCambridgeUnited Kingdom; ^2^ Duke University School of MedicineDurhamNorth CarolinaUSA; ^3^ Addenbrooke's Hospital Department of OtolaryngologyCambridgeUnited Kingdom; ^4^ Department of Medical PhysicsMemorial Sloan Kettering Cancer CenterNew YorkNew YorkUSA; ^5^ Addenbrooke's Hospital Department of RadiologyCambridgeUnited Kingdom; ^6^ Department of Epidemiology and BiostatisticsMemorial Sloan Kettering Cancer CenterNew YorkNew YorkUSA; ^7^ Department of RadiologyMemorial Sloan Kettering Cancer CenterNew YorkNew YorkUSA; ^8^ Department of SurgeryMemorial Sloan Kettering Cancer CenterNew YorkNew YorkUSA; ^9^ Department of MedicineMemorial Sloan Kettering Cancer CenterNew YorkNew YorkUSA; ^10^ Cambridge Teaching Hospitals ENT DepartmentCambridgeUnited Kingdom

**Keywords:** textural analysis, diffusion‐weighted MRI, thyroid tumors

## Abstract

**Purpose:**

Ultrasound‐guided fine needle aspirate cytology fails to diagnose many malignant thyroid nodules; consequently, patients may undergo diagnostic lobectomy. This study assessed whether textural analysis (TA) could noninvasively stratify thyroid nodules accurately using diffusion‐weighted MRI (DW‐MRI).

**Methods:**

This multi‐institutional study examined 3T DW‐MRI images obtained with spin echo echo planar imaging sequences. The training data set included 26 patients from Cambridge, United Kingdom, and the test data set included 18 thyroid cancer patients from Memorial Sloan Kettering Cancer Center (New York, New York, USA). Apparent diffusion coefficients (ADCs) were compared over regions of interest (ROIs) defined on thyroid nodules. TA, linear discriminant analysis (LDA), and feature reduction were performed using the 21 MaZda‐generated texture parameters that best distinguished benign and malignant ROIs.

**Results:**

Training data set mean ADC values were significantly different for benign and malignant nodules (*P* = 0.02) with a sensitivity and specificity of 70% and 63%, respectively, and a receiver operator characteristic (ROC) area under the curve (AUC) of 0.73. The LDA model of the top 21 textural features correctly classified 89/94 DW‐MRI ROIs with 92% sensitivity, 96% specificity, and an AUC of 0.97. This algorithm correctly classified 16/18 (89%) patients in the independently obtained test set of thyroid DW‐MRI scans.

**Conclusion:**

TA classifies thyroid nodules with high sensitivity and specificity on multi‐institutional DW‐MRI data sets. This method requires further validation in a larger prospective study. Magn Reson Med, 2015. © 2015 The Authors. Magnetic Resonance in Medicine published by Wiley Periodicals, Inc. on behalf of International Society for Magnetic Resonance in Medicine. This is an open access article under the terms of the Creative Commons Attribution License, which permits use, distribution and reproduction in any medium, provided the original work is properly cited. **Magn Reson Med 75:1708–1716, 2016. © 2015 The Authors. Magnetic Resonance in Medicine published by Wiley Periodicals, Inc. on behalf of International Society for Magnetic Resonance.**

## INTRODUCTION

Thyroid cancer is the most common malignant endocrine tumor, with an annual incidence in the United States of 12.2 per 100,000 in men and women per year 1. Thyroid nodules may have benign or malignant pathology and are diagnosed before surgery using ultrasound‐guided fine needle aspirate cytology (FNAC), the current gold standard. Thyroid nodules are common and ultrasound is an excellent screening tool to determine which nodules require FNAC. Despite repeated aspirates, however, up to 7% of nodules yield nondiagnostic cytology, classified as Thy1 2. A further 15%–30% of FNACs represent an indeterminate cytology (Thy3), where a follicular or Hurthle cell neoplasm is reported 3. The risk of malignancy within these Thy1 and Thy3 indeterminate nodules is 20%–30% 4. These cytological categories with management recommendations are shown in Table 1.

**Table 1:** Thyroid Nodule Cytology Classification Schema According to the 2007 British Thyroid Association Guidelines

	Thy1	Thy2	Thy3	Thy4	Thy5
Definition	Nondiagnostic/cysts	Nonneoplastic	Indeterminate	Suspicious for malignancy	Malignant
Current management recommendations	Repeat FNAC and ultrasonography at follow‐up	Repeat FNAC 3–6 months	Diagnostic lobectomy	Repeat FNAC, then either diagnostic lobectomy or radical treatment	Radical treatment

A thyroid lobectomy may be therapeutic for Thy3 (indeterminate) patients if the histology is benign. However, if a malignant diagnosis is made, patients are likely to need completion thyroidectomy with central compartment lymph node dissection followed by radioiodine therapy. Accurate preoperative diagnosis would therefore improve surgical planning as well as reduce unnecessary operations, since patients with malignant tumors would receive one definitive operation. Thus, more research is needed to explore new modalities that discriminate between malignant and benign thyroid nodules.

Recent interest has centered on DW‐MRI, which measures the apparent diffusivity of tissue water. When diffusion‐sensitizing magnetic gradients are applied, Brownian motion of water protons creates a DW‐MRI signal that can be used to generate maps of the apparent diffusion coefficient (ADC). Diffusion measurements can provide insight into tissue structure and organization, and can discriminate between benign and malignant tumors in organs such as the breast, liver, and uterus 5. It is hypothesized that because of the increased cell proliferation in malignant tumors, water protons undergo less Brownian motion, thus lowering ADC. Several recent studies of thyroid nodules in small cohorts of patients have supported this hypothesis, as delineated in Table 2 6, 7, 8, 9, 10, 11, 12.

**Table 2:** Comparison of Thyroid Tumor DW‐MRI Studies

Study/Tissue Type	n	Mean ADC (×10^−3^ mm^2^/s) ± SD	Optimum ADC Threshold
Razek et al. 6			0.98 × 10^−3^ mm^2^/s
Benign			
Adenomatous nodule	42	1.8 ± 0.14	
Follicular adenoma	6	1.7 ± 0.17	
Cyst	8	1.9 ± 0.38	
Malignant			
Papillary	4	0.68 ± 0.23	
Follicular	3	0.77 ± 0.17	
Bozgeyik et al. 7			0.62 × 10^−3^ mm^2^/s
Benign	88	1.15 ± 0.43	
Malignant	5	0.30 ± 0.20	
Schueller‐Weidekamm et al. 8			2.25 × 10^−3^ mm^2^/s
Benign	20	1.93 ± 0.25	
Malignant	5	2.73 ± 0.65	
Contralateral	20	1.44 ± 0.65	
Erdem et al. 9			NA
Benign	52	2.75 ± 0.60	
Malignant	9	0.70 ± 0.31	
Control normal	24	1.34 ± 0.28	
Nakahira et al. 10			1.60 × 10^−3^ mm^2^/s
Benign	23	1.93 ± 0.37	
Malignant	19	1.20 ± 0.25	
Mutlu at al. 11			1.60 × 10^−3^ mm^2^/s
Benign	46	1.6 ± 0.1	
Malignant	5	0.8 ± 0.2	
Dilli et al. 12			NA
Benign	40	1.98 ± 0.48	
Malignant	19	0.83 ± 0.18	

Abbreviations: NA, not available; SD, standard deviation.

Textural analysis (TA) has become an attractive clinical tool, as it quantifies pixel intensity variation otherwise invisible to the naked eye and thus aids in characterizing underlying tissue structures. Several TA studies have shown good discrimination of thyroid nodules on ultrasound images 13, 14, 15 and better distinction between benign and malignant thyroid lesions on nuclear chromatin images 16, but none have used TA on DW‐MRI scans of the thyroid. The aim of this study was to assess whether textural analysis could improve the accuracy, sensitivity, and specificity of DW‐MRI for the stratification of malignancy in suspicious thyroid nodules.

## METHODS

Two cohorts of patients, from the Cambridge University Foundation Hospital Trust, UK (Cambridge) and Memorial Sloan Kettering Cancer Center, USA (MSKCC) were included in this multi‐institutional study. The clinical protocols and methods of analysis at each institution are described below.

### Training Data Set, Cambridge University Hospitals Foundation Trust, UK

#### Study Design and Patient Population

A total of 42 patients (11 men, mean age 57.1 y [range, 29–79 y]; 31 women, mean age 42.9 years [range, 18–78 y]) with a preoperative cytological status that was indeterminate (Thy3), suspicious (Thy4), or diagnostic of thyroid cancer (Thy5) were prospectively recruited into this pilot study between February 2010 and January 2012, following ethical approval granted by the Local Research Ethics Committee in January 2010. The inclusion criteria for the study were: 1) proven Thy3–Thy5 thyroid lesions on cytological classification; 2) a follicular neoplasm, suspected malignancy, or an inconclusive lesion on ultrasound‐guided thyroid core biopsy; and 3) a plan for surgical excision. Exclusion criteria included the typical contraindications to MR imaging. Initially, FNAC or core biopsy was performed on all nodules and reported by an experienced cytologist or pathologist. Next, patients underwent preoperative MRI (protocol below). Two patients then opted out of surgical treatment and were excluded from the study. The remaining 40 patients underwent thyroid surgery. The type of thyroid surgery depended on the recommendation of the local thyroid multidisciplinary team meeting, which followed the 2007 British Thyroid Association Guidelines (see Table 1). The postoperative histology and nodule dimensions for the 40 patients who underwent surgery were reported by an experienced pathologist and correlated to the preoperative images.

#### MRI Protocol

MRI studies were conducted with a 3T HDx scanner (GE Healthcare, Waukesha, Wisconsin, USA). Signals were transmitted using a body coil and were received using two channels of a four‐channel phased array surface coil (Machnet BV, Elde, The Netherlands) designed for studies of the carotid arteries. One arm of the coil was centered over the area of interest (thyroid nodule) to maximize local sensitivity and secured by a soft cervical collar to reduce motion artifact. After a three‐plane localizer, the following sequences were performed:

1. Fast spin echo axial T_1_: echo time (TE) = 12 ms, repetition time (TR) = 580 ms, field of view (FOV) = 18 cm, matrix = 256 × 192, number of averages = 4, and number of slices = 15 (slice thickness = 5 mm, spacing = 1 mm); scan duration = 2 min, 31 s.
2. Fast spin echo axial T_2_: TE = 102 ms, TR = 3780 ms, FOV = 18 cm, matrix = 384 × 256, number of averages = 2, and number of slices = 15 (slice thickness = 5 mm, spacing = 1 mm); scan duration = 1 min, 38 s.
3. Fast spin echo axial T_2_ with fat saturation: same as sequence 2, except TR = 3360 ms, matrix 320 × 192, and a chemical shift selective fat suppression pulse was used; scan duration = 1 min, 13 s.
4. Diffusion‐weighted dual spin echo planar imaging (DW‐EPI): TE = 81 ms, TR = 2200 ms, FOV = 22 cm, matrix = 128 × 128, and number of averages = 16 (slice thickness = 5 mm, spacing = 1 mm); scan duration = 2 min, 21 s; b values of 0 and 500 s/mm^2^ were acquired.

Fat saturation was achieved using both a spectrally selective saturation pulse and a water‐selective excitation pulse. Spatial saturation bands were also used to remove signal from overlying fat and other nearby tissues. The scanner software automatically interpolated the images to a reconstructed matrix of 256 × 256 by zero‐filling k‐space.

#### Image Analysis

The ADC maps were calculated by fitting the signal intensities in the images with b values of 0 and 500 s/mm^2^ as follows:

(1) S(500)=S(0) × exp(−500 × ADC)

An experienced neuroradiologist who was blinded to the clinical data of the subjects drew regions of interest (ROIs) around the thyroid lesions on each image slice containing a lesion, avoiding any obvious cysts or hematomata from previous biopsy. ROI measurements were defined on ADC maps, with reference to the T_2_‐weighted images, using an Advantage Windows workstation and FuncTool software (GE Healthcare). Images where a thyroid nodule was not clearly identified (due to the small volume of noncystic tissue sampled or to the severity of DW‐MRI–related distortions) were excluded from analysis. Sixteen patients were excluded due to withdrawal from surgery (n = 2), image distortion (n = 4), nodule too small to be identified (<10 mm) (n = 3), and cystic nodule (n = 7), leaving 26 patients with reliable images for analysis. In this cohort, there were 10 patients with malignant nodules and 16 patients with benign pathology. To maintain consistency with the test data set, which was a population of exclusively papillary carcinomas, the malignant nodules in the local data set were limited to the eight cases of papillary carcinoma. A total of 24 patients with 94 image slices were included in the final training set for analysis. The number of image slices per patient ranged from 1 to 7 in the training set (mean = 4).

The mean ADC values for each slice in the nodule were derived using FuncTool software. The mean ADCs from multiple slices were then pooled as follows:

(2) $$\bar{x}=\frac{w_{1}x_{1}+w_{2}x_{2}+\cdots +w_{n}x_{n}}{w_{1}+w_{2}+\cdots +w_{n}}$$

where $\bar{x}$ is the overall weighted‐mean ADC, $w_{1}$ is the area of the first ROI, $x_{1}$ is the mean ADC of the first ROI, $w_{2}$ is the area of the second ROI, $x_{2}$ is the mean ADC of the second ROI, and so forth.

#### Statistical Analysis

Weighted‐mean ADC values were plotted against postoperative histology (benign and malignant thyroid tissue) and the ROI areas and 95% confidence intervals (CIs) were calculated using GraphPad Prism (version 5.00 for Windows; GraphPad Software, San Diego, California, USA). A two‐sample *t* test was used to compare mean values between benign and malignant cases.

### Test Data Set, Memorial Sloan Kettering Cancer Center

#### Study Design and Patient Population

Between January 2011 and March 2012, a convenience sample of 25 adult patients (≥18 years) undergoing surgical consultation for thyroidectomy on the basis of a thyroid nodule FNAC either 1) demonstrating papillary thyroid cancer or 2) suspicious for thyroid cancer were enrolled in a prospective clinical trial evaluating multiparametric MRI including DW‐MRI in the preoperative evaluation of head and neck tumors. The prospective protocol was approved by the MSKCC local institutional review board. After providing appropriate informed consent, all subjects underwent research MRI prior to thyroid surgery. The exclusion criteria were 1) presence of contraindication to MRI, 2) tumor size >5 cm (as detected by ultrasonography), and 3) claustrophobia. Of the 25 patients initially enrolled in the study, seven patients were excluded from the study due to either distorted image quality (n = 5) or small tumor size such that visualization was difficult on DW‐MRI images (n = 2). Eighteen patients were suitable for the final analysis.

#### MRI Protocol

MRI examination was performed on a 3T HDx scanner (GE Healthcare) using an eight‐channel neurovascular phased‐array coil. The MRI study consisted of standard multiplanar (sagittal, axial, coronal) T_1_‐ and T_2_‐weighted imaging scans followed by DW‐MRI scans. The duration of the entire examination was approximately 30 min.

The T_1_‐ and T_2_‐weighted MRI scans covered the whole thyroid gland with a slice thickness of 5 mm, FOV of 20–24 cm, and acquisition matrix of 256 × 256. For the T_1_‐weighted MRI, the TR and TE were 500 ms and 15 ms, respectively; for the T_2_‐weighted MRI, the TR and TE were 4000 ms and 80 ms, respectively.

DW‐MRI data were acquired using a single‐shot EPI spin echo sequence (TR = 4000 ms; TE = 98–104 ms; number of excitations = 4; 3 orthogonal directions) with b values of 0 and 500 s/mm^2^. Fat suppression, shimming (shimming FOV = 14–16 cm), and parallel imaging (acceleration factor = 2) techniques were used. The DW‐MRI scans were focused on thyroid tumors using the following parameters: number of slices = 4–8, slice thickness = 5 mm, gap = 0 mm, FOV = 20–24 cm, and acquisition matrix = 128 × 128 (zero‐filled and reconstructed to 256 × 256 pixels). Images were all obtained in axial planes.

#### Image Analysis

The ROIs for papillary thyroid cancers were placed within the thyroid gland images avoiding obvious cystic, hemorrhagic, or calcified portions. Based on the radiological and clinical information including ultrasound reports, they were drawn on the DW‐MR images by a neuroradiologist who had more than 10 years of experience. The ROI encompassed the entire nodule of interest with a minimum two‐dimensional ROI considered to be 17 mm^2^ (ie, 17 pixels). The ADC values were calculated using Equation (1) with b values of 0 and 500 s/mm^2^. A noise floor rectification scheme was used in the ADC calculation 17, which was performed on a voxel‐by‐voxel basis, generating an ADC map as well as averaged values for the ROIs.

### Textural Analysis

**Figure 1:**
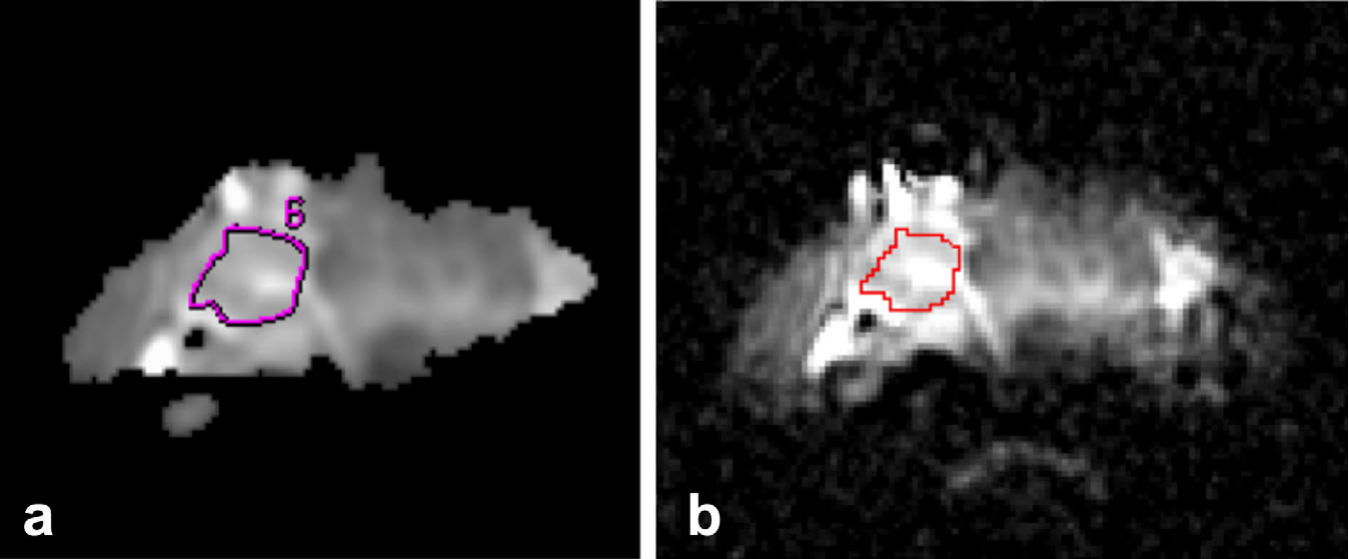
ADC images for a patient with a follicular adenoma from the training set. (**a**) Neuroradiologist‐defined ROI of the lesion on a bitmap‐format ADC map in FuncTool. (**b**) The same ROI shown on the original resolution DICOM‐format ADC map in ImageJ.

Textural analysis (TA) was performed using MaZda (Institute of Electronics, Technical University of Łódź, Wólczańska, Poland), a freely available software package 18, 19, 20. Two‐dimensional ROIs delineated by radiologists at each institution were transferred to MaZda by using binary masks in ImageJ (National Institutes of Health, Bethesda, Maryland, USA). An example of the ROI transfer process is shown in Fig. 1.

### Training Data Set Analysis

The MaZda textural analysis resulted in a report with more than 300 texture parameters for each ROI in the training data set. There were seven texture feature categories included in this analysis: run‐length matrix, wavelet transform, gradient, geometric, histogram, and autoregressive model parameters in addition to features derived from co‐occurrence matrices in four directions (0°, 45°, 90°, and 135°) at pixel pair distances ranging from 1 to 5 pixels in separation. Feature reduction was necessary to reduce the dimensionality. MaZda offers three feature reduction algorithms: mutual information, Fisher coefficient, and classification error probability and average correlation coefficients (POE + ACC). Each algorithm determined the 10 texture features that best distinguished the selected classes in the program (eg, benign and malignant), such that a combined total of up to 30 parameters were identified for further investigation 21, 22, 23. This dimensionality was further reduced by exporting the selected features into the statistical package b11 (Institute of Electronics, Technical University of Łódź). Within b11, subsets of the top 30 parameters were further evaluated by sequentially eliminating features of lower significance based on the MaZda‐assigned rank (eg, top 29, top 28, top 27, etc., down to the top two parameters). The misclassification rate for distinguishing benign and malignant nodules using linear discriminant analysis (LDA) for each of these subsets was then observed. The final subset achieving the lowest misclassification rate was selected for the LDA model. The resultant most discriminant factor 1 (MDF1) values in the LDA model of the training set were exported into GraphPad Prism to determine the sensitivity and specificity of the selected cutoff MDF1 value and to generate a receiver operator characteristic (ROC) curve. Additional analysis included comparing the number of central slices and end slices that were misclassified in nodules containing at least three slices, and classifying thyroid nodules on the basis of the slice containing the lowest MDF1 value (lowest scoring slice). The lowest scoring slice was considered rather than the highest scoring slice in order to minimize false positive results.

#### Test Data Set Analysis

The DW‐EPI images and ROIs of the test data set were imported into MaZda and processed in the same way as the training set to generate >300 texture features per ROI using the same seven texture classes as were considered for the training set. Next, the MDF1 was calculated using the same LDA model equation and final subset of parameters used for the training set. The resultant MDF1 values were used to classify the test set samples into either malignant or benign categories, based on the predefined training set MDF1 cutoff value. The additional comparisons of central versus end slice misclassification rates and lowest scoring slice analysis as described in the prior section were also performed.

## RESULTS

### Training Data Set

The T_2_‐weighted and DW‐EPI images were collected in 40 patients and achieved sufficient quality for reliable ROI definition in 26 patients with a variety of benign and malignant tumor subtypes. Fig. 1 depicts an example of one patient's ADC maps with ROIs drawn avoiding a cystic area. Each ROI was originally delineated by an experienced neuroradiologist using the FuncTool software (GE Healthcare) and subsequently carefully traced using ImageJ software onto the original resolution ADC maps so that binary masks of these ROIs could be imported into MaZda to preserve the original ROI locations. For each patient, the entire nodule was classified as benign or malignant on the basis of histological analysis. The maximum nodule diameter was determined, with a mean and standard deviation of 29.3 ± 8.0 mm for the benign nodules and 33.3 ± 10.4 mm for the malignant nodules.

**Figure 2:**
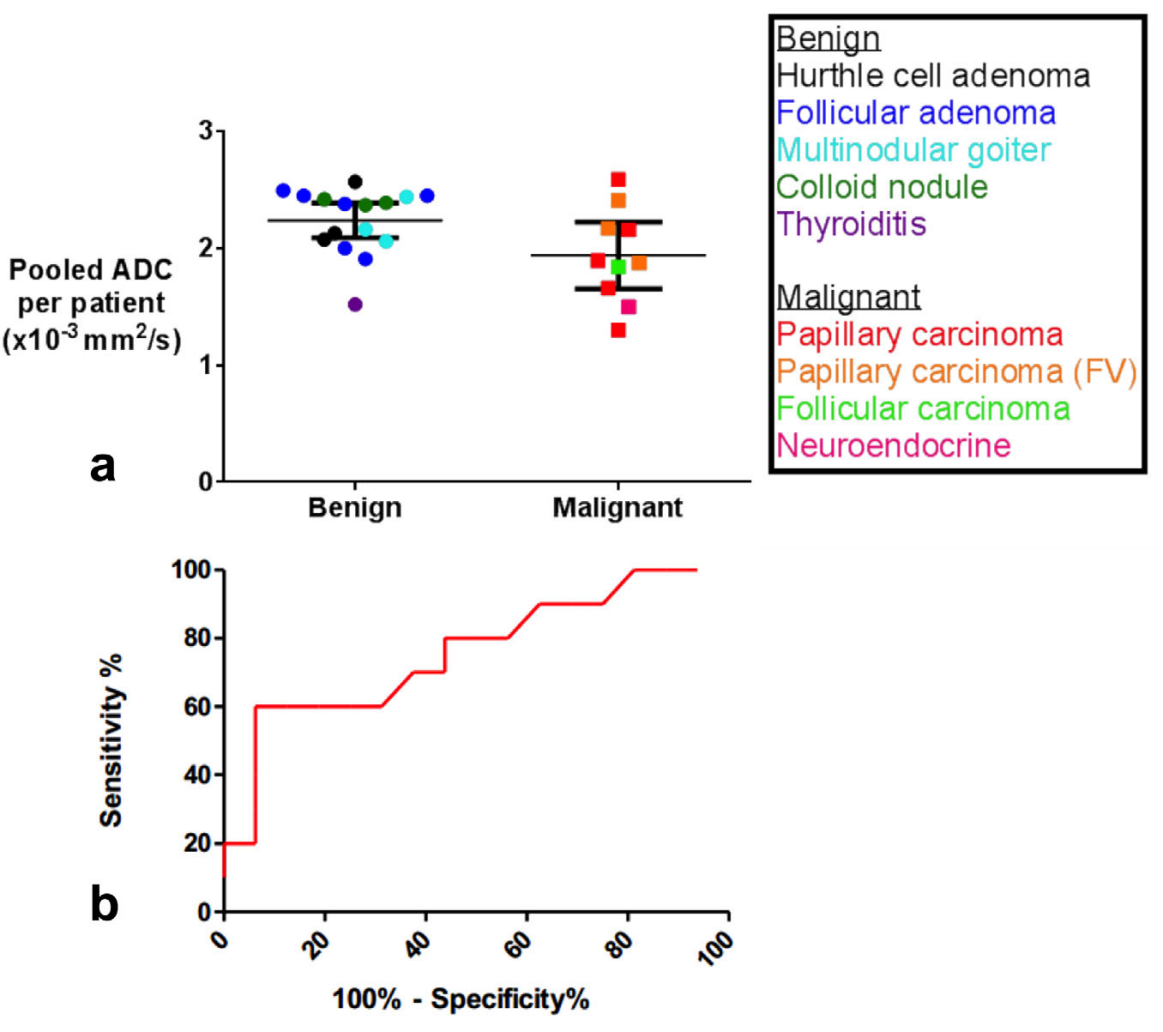
(a) Overall weighted mean and 95% CI of the ADC values of benign and malignant thyroid tumors for DW‐EPI (*P* = 0.02 for the difference between means). The overall weighted mean ADC for benign tumors was 2.24 × 10^−3^ mm^2^/s (95% CI, 2.09–2.39), and for papillary carcinoma malignant tumors it was 1.92 × 10^−3^ mm^2^/s (95% CI, 1.65–2.19). The follicular carcinoma (n = 1) and neuroendocrine (n = 1) tumors shown in this graph were not included in the final analysis. (**b**) ROC curve for performance of ADC using a cutoff value of 2.16 × 10^−3^ mm^2^/s to distinguish benign and malignant nodules demonstrates an AUC of 0.73 (95% CI, 0.51–0.95), sensitivity of 70%, and specificity of 63%.

The performance of ADC alone in distinguishing malignant and benign nodules was determined. Fig. 2 shows the overall weighted‐mean ADC values for benign and malignant tumors, with means for each patient and corresponding subtype of thyroid nodule. The overall weighted‐mean ADC for benign tumors was 2.24 × 10^−3^ mm^2^/s (95% CI, 2.09–2.39) and for malignant tumors was 1.92 × 10^−3^ mm^2^/s (95% CI, 1.65–2.19). The difference between the means of the benign and malignant nodules was significant (*P* = 0.02); however, there was overlap between the CIs, resulting in an area under the curve (AUC) of 0.73 (95% CI, 0.51–0.95), sensitivity of 70%, and specificity of 63% on ROC analysis using a cutoff ADC value of 2.16 × 10^−3^ mm^2^/s.

**Figure 3:**
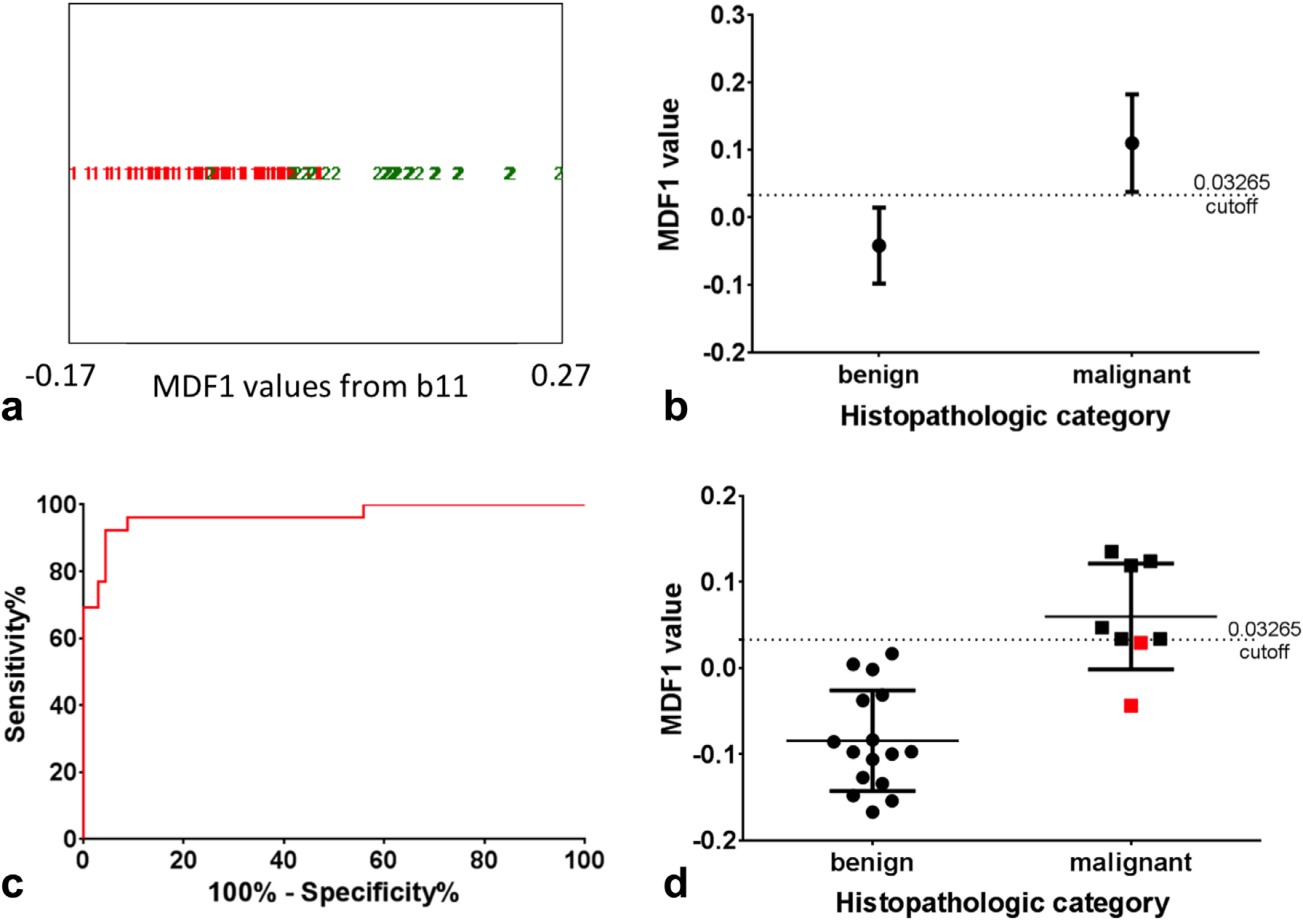
Texture‐based classification of individual images (a‐c) and the nodule as a whole (d). (**a**) Output from b11 for the LDA classification MDF1 values for all 94 slices of the training set. MDF1 values are shown for benign and malignant slices, where the red 1 symbol = benign and the green 2 symbol = malignant. Eighty‐nine of the 94 slices were classified correctly using a cutoff value of 0.03265. (**b**) Mean and standard deviation of the benign and malignant MDF1 values. (**c**) ROC curve for using this MDF1 cutoff as a diagnostic tool (*P* < 0.0001 and AUC of 0.97 [95% CI, 0.92–1.0]). (**d**) LDA classification results for the slice with the lowest MDF1 value per patient (lowest scoring slice analysis). Twenty‐two of the 24 nodules were classified correctly using the same training set cutoff value of 0.03265. The mean and standard deviation values are shown along with separate points for each nodule. The two misclassified nodules were both malignant and are shown in red.

For the training set malignant category, only nodules containing papillary carcinoma were included. Texture analysis on the DW‐EPI images yielded higher sensitivity and specificity values (Fig. 3) than the ADC analysis. Table 3 lists the original top 30 MaZda texture analysis parameters obtained by using the three feature‐reduction algorithms (mutual information, Fisher coefficient, and POE + ACC), the final subset of the top 21 parameters used for the LDA model, and the corresponding texture classes for each parameter. This texture analysis LDA model used a cutoff MDF1 value of >0.03265 as the basis for classification as malignant. It correctly classified 89 of 94 thyroid nodule slices in the training set, resulting in a misclassification rate of 5.3%, an area under the curve (AUC) of 0.97 (95% CI, 0.92–1.0), and the sensitivity and specificity values were 92% and 96%, respectively. Of the five misclassified slices, one was a central slice (slice 2 of 7) and four were end slices (either the first or last slice). Distinguishing whole thyroid nodules on the basis of the slice per nodule with the lowest MDF1 value (lowest scoring slice) resulted in correct classification of 22/24 nodules in the training set based on the predefined cutoff value (Fig. 3).

**Table 3:** Top 30 Texture Parameters and Top 21 Feature Subset for Thyroid Stratification Model

MaZda Rank	Texture Class	Top 30 Texture Parameters	Top 21 Feature Subset
1	Geometric	GeoY	GeoY
2	Geometric	GeoX	GeoX
3	Co‐occurrence matrix	S(0,3)SumAverg	S(0,3)SumAverg
4	Co‐occurrence matrix	S(0,4)SumAverg	S(0,4)SumAverg
5	Co‐occurrence matrix	S(0,1)SumAverg	S(0,1)SumAverg
6	Co‐occurrence matrix	S(0,2)SumAverg	S(0,2)SumAverg
7	Co‐occurrence matrix	S(0,5)SumAverg	S(0,5)SumAverg
8	Co‐occurrence matrix	S(2,0)SumOfSqs	S(2,0)SumOfSqs
9	Co‐occurrence matrix	S(1,0)SumOfSqs	S(1,0)SumOfSqs
10	Co‐occurrence matrix	S(2,2)Correlat	S(2,2)Correlat
11	Geometric	GeoM2xy	GeoM2xy
12	Co‐occurrence matrix	S(1,0)SumVarnc	S(1,0)SumVarnc
13	Co‐occurrence matrix	S(3,‐3)DifVarnc	S(3,‐3)DifVarnc
14	Geometric	GeoS2	GeoS2
15	Geometric	GeoXYo	GeoXYo
16	Autoregressive model	Teta1	Teta1
17	Co‐occurrence matrix	S(2,0)SumAverg	S(2,0)SumAverg
18	Geometric	GeoYo	GeoYo
19	Wavelet transform	WavEnHH_s‐3	WavEnHH_s‐3
20	Co‐occurrence matrix	S(5,5)DifEntrp	S(5,5)DifEntrp
21	Co‐occurrence matrix	S(1,0)SumAverg	S(1,0)SumAverg
22	Co‐occurrence matrix	S(1,1)SumAverg	
23	Wavelet transform	WavEnLL_s‐3	
24	Co‐occurrence matrix	S(2,2)SumAverg	
25	Co‐occurrence matrix	S(1,‐1)SumAverg	
26	Co‐occurrence matrix	S(2,‐2)SumAverg	
27	Co‐occurrence matrix	S(3,0)SumAverg	
28	Co‐occurrence matrix	S(3,3)SumAverg	
29	Co‐occurrence matrix	S(4,0)SumAverg	
30	Co‐occurrence matrix	S(3,‐3)SumAverg	

#### Test Data Set

**Figure 4:**
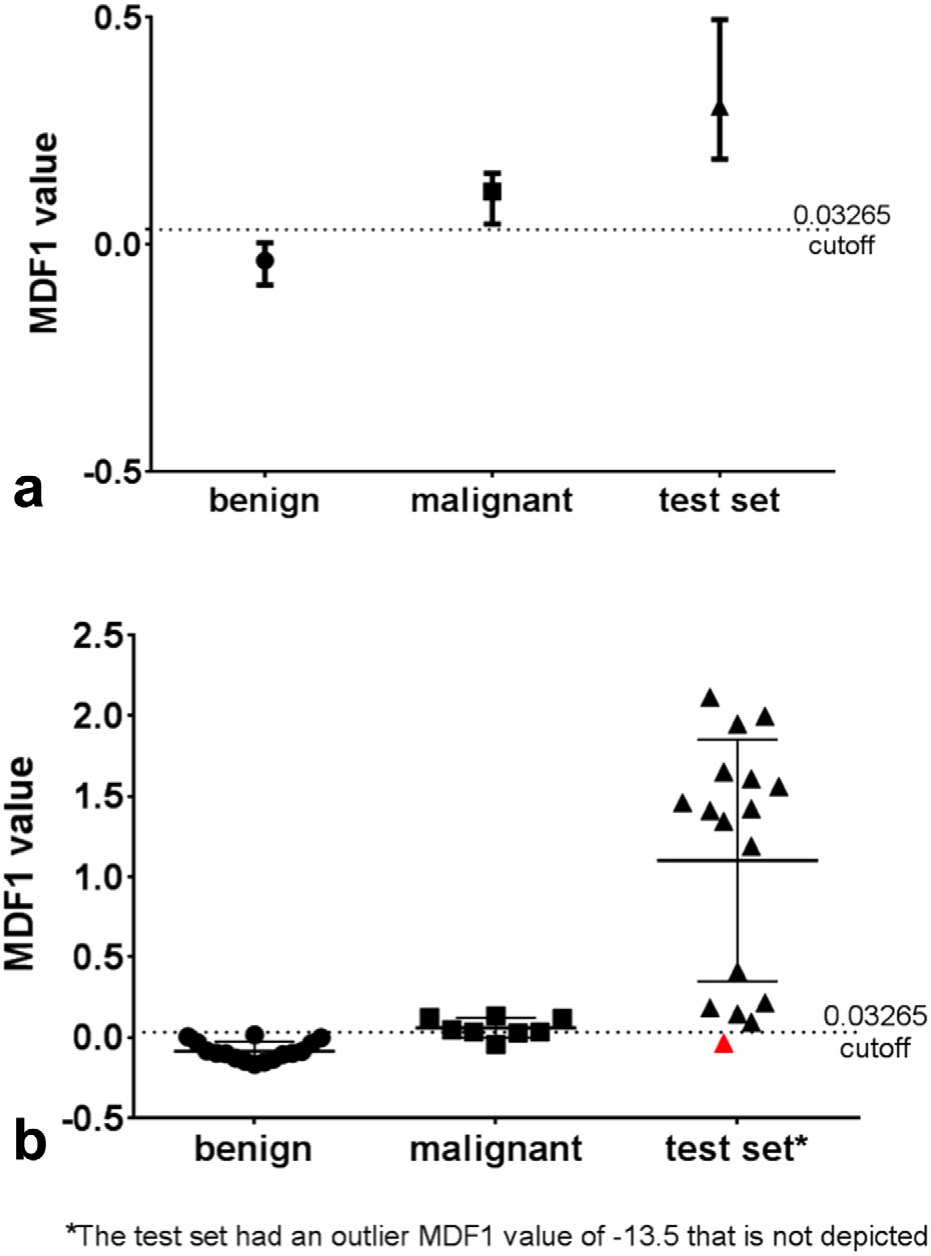
(a) LDA classification most discriminant factor 1 (MDF1) results for all 34 slices of the test set, with median and interquartile ranges displayed alongside training set results. Thirty‐two of the 34 slices were classified correctly using the training set cutoff value of 0.03265. (**b**) LDA classification results for the slice with the lowest MDF1 value per patient (lowest scoring slice analysis) for the test set. Sixteen of 18 nodules were classified correctly using the same training set cutoff value of 0.03265 for MDF1 values. Mean and standard deviation values are shown along with separate points for each nodule. One of the two misclassified test set nodules is shown in red, and the other was an outlier (data not shown; MDF1 value = −13.5).

Our LDA model was tested on an independent data set from MSKCC containing papillary carcinoma thyroid nodules. The mean ADC value for this cohort was 1.80 × 10^−3^ mm^2^/s (95% CI, 1.52–2.08). Using the same 21 texture parameters from the training set LDA model, 32/34 slices were classified correctly, resulting in an overall misclassification rate of 5.9% (Fig. 4). Using the same cutoff MDF1 value of the training set (>0.03265), this resulted in a sensitivity of 89% (95% CI, 65–99) and specificity of 97% (95% CI, 74–100). Of the two misclassified slices, one was a central slice (2 of 3) and one was an end slice. The lowest scoring slice analysis correctly classified 16/18 nodules in the test set (Fig. 4).

## DISCUSSION

### Comparisons with Other Studies

Our results comparing benign and malignant ADCs are consistent with recent reports, as shown in Table 2 6, 7, 8, 9, 10, 11, 12. All of these reports except one 8 found lower ADCs in malignant thyroid nodules compared with benign nodules, supporting the hypothesis that increased cellularity and reduced extracellular extravascular space restrict water diffusion in malignant nodules 24. However, our results indicated poor sensitivity and specificity for using ADC alone to discriminate benign and malignant pathology. This could be due to cytological similarities, because both malignant and benign follicular thyroid tumors may be well‐differentiated and exhibit significant cytological overlap 25. Additionally, in our study some small cystic and necrotic areas may have been included in the ROIs despite efforts to avoid them, which would have artifactually increased the mean ADC value of the nodule.

### Strengths of the Study

To our knowledge, this is the first attempt to use texture analysis (TA) for diffusion‐weighted imaging of suspected thyroid tumor nodules. Validating this model on an independent data set from another institution provides additional evidence that this tool can be implemented in a clinical setting and is robust against institutional differences in imaging equipment and technique. Our study demonstrates very high performance for both the training and test data sets as evidence of this robustness.

### Limitations of DW‐MRI Results

The DW‐MRI images showed distortion at 3T, and, based on neuroradiologist exclusion criteria, only 26/40 patients (University of Cambridge) and 18/25 patients (MSKCC) had images that could be interpreted. MSKCC excluded seven patients due to either distorted image quality (n = 5) or small tumor size resulting in poor visualization on DW‐MRI images (n = 2). Of note, seven cystic nodules (University of Cambridge) were excluded. However, other common thyroid imaging techniques such as ultrasound elastography are also unable to image cystic nodules 26. Better pulse sequences are necessary to reduce image distortion and improve interpretability such that radiologists are able to draw reliable ROIs around small nodules. One potential method that merits further investigation is reduced FOV DW‐EPI 27, which has previously shown less distortion in diffusion imaging of the kidneys 28.

### Possible Methodological Improvements

The small sample size of this study (24 patients in the training set [University of Cambridge], 18 in the test set [MSKCC]) results in underrepresentation of several tumor subtypes. Moreover, our decision to limit the malignant pathology in our training set to only papillary carcinomas reduces its universal applicability to distinguish benign nodules from other types of malignant pathology. A larger study is required, including all common tumor pathologies.

Another concern is that the large number of texture parameters used for the LDA model may “overfit” the training set, as the 21 parameters were combined into a linear discriminant analysis model to represent the 94 slices in the training set. To reduce the risk of overfitting, the top 30 parameters from the three feature reduction tools of the original MaZda output were further examined in subsets in an attempt to reduce the dimensionality of the texture parameters while still achieving the lowest misclassification rate; that resulted in the number of texture features being reduced from 30 to 21 parameters. It is encouraging that 32/34 slices in the independently obtained test set from another institution were classified correctly. However the number of parameters is still quite large relative to the size of the data set; therefore, overfitting remains a risk. Testing this tool on larger data sets will better characterize its robustness.

One potential technical concern is that the image resolution of 256 × 256, obtained after scanner software zero‐fill interpolation, may alter the image's textural properties when compared with the original images in which the resolution was determined by pulse sequence parameters (128 × 128). Previous studies have shown that zero‐fill interpolated images enhance the textural differences of physically distinct structures 29, 30. However, it is routine clinical practice to use MR scanner software to interpolate images by automatic zero‐filling of k‐space to achieve a resolution of 256 × 256. Thus, our results are reflective of results obtained using routine clinical images.

An additional technical consideration is the difference in TR between the diffusion MRI sequences used at the two institutions. Variations in TE, TR, and other pulse sequence variables have been shown to affect texture features in phantom studies 31. Thus, the variation in TR values used at the two institutions may impact the quality of the textural calculations; however, this concern may be partially alleviated by the dominance of co‐occurrence matrix‐derived texture parameters in our LDA model. Co‐occurrence‐based features were found to be the most robust of the texture categories examined by Mayerhoefer et al. 31. This finding has been corroborated by recent studies that identified co‐occurrence matrix features as superior to all other texture classes in distinguishing benign and malignant breast lesions 32 and certain co‐occurrence matrix features as helpful in differentiating brain malignancies 33. Therefore, although MRI acquisition parameters certainly need to be taken into account in further clinical applications of this technique, it is encouraging that our model is primarily comprised of the co‐occurrence features previously deemed robust in multiple clinical studies.

Furthermore, the low success rate of 26/40 reliable ROIs (Cambridge data set) will not apply in future studies, since small and cystic lesions (n = 10 in the training data set) could be identified by standard ultrasound, and patients with inappropriate lesion characteristics would not be offered the DW‐MRI test. Patients excluded from this study on the grounds of poor or distorted image quality (n = 4 training set, n = 5 test set) present another challenge. However, we anticipate that this problem will also be greatly reduced in the future due to the development of DW‐MRI techniques with reduced distortion 27. In principle, if thinner slices were analyzed, the three‐dimensional TA capability of MaZda could also improve the classification, as patterns in the *z*‐axis direction could be detected.

In conclusion, our pilot study indicates the potential for textural analysis to be used on DW‐MRI images for noninvasively categorizing the malignancy of thyroid nodules in a single, definitive procedure, thus sparing patients from unnecessary operations and waiting times associated with a diagnostic lobectomy. The current multicenter study shows promise for the limited patient population represented by our investigations. The ability of the LDA method to classify images obtained in another institution using different imaging parameters suggests that it will be robust. A larger, prospective study is now needed to fully prove this model.
